# Microanatomic analyses of extratemporal facial nerve and its branches, hypoglossal nerve, sural nerve, and great auricular nerve

**DOI:** 10.1016/j.bjorl.2021.06.006

**Published:** 2021-07-20

**Authors:** Ibrahim Erdim, Veysel Gurbuz, Emrah Sapmaz, Selcuk Cetin, Fikret Gevrek

**Affiliations:** a Tokat Gaziosmanpasa University, Health Education and Training Hospital, Otorhinolaryngology Department, Tokat, Turkey; b Turkey Republic the Ministry of Justice, Tokat Forensic Medicine Department, Tokat, Turkey; c Tokat Gaziosmanpasa University, Health Education and Training Hospital, Forensic Medicine Department, Tokat, Turkey; d Tokat Gaziosmanpasa University, Medical Faculty, Histology Deparment, Tokat, Turkey

**Keywords:** Facial, Hypoglossal, Sural, Great auricular, Histomorphometry

## Abstract

**Objective:**

To investigate microanatomic organizations of the extratemporal facial nerve and its branches, hypoglossal nerve, sural nerve, and great auricular nerve.

**Methods:**

Nerve samples were dissected in 12 postmortem autopsies, and histomorphometric analyses were conducted.

**Results:**

There was no significant difference between the right and left sides of the nerve samples for the nerve area, fascicle area, number of fascicles and average number of axons. The lowest mean fascicle number was found in the hypoglossal nerve (4.9 ± 1.4) while the highest was in great auricular nerve (11.4 ± 6.8). The highest nerve area (3,182,788 ± 838,430 μm^2^), fascicle area (1,573,181 ± 457,331 μm^2^) and axon number (14,772 ± 4402) were in hypoglossal nerve (*p* < 0.05). The number of axons per unit nerve area was higher in the facial nerve, truncus temporofacialis, truncus cervicofacialis and hypoglossal nerve, which are motor nerves, compared to the sural nerve and great auricular nerve, which are sensory nerves (*p* < 0.05). The number of axons per unit fascicle area was also higher in motor nerves than in sensory nerves (*p* < 0.05).

**Conclusion:**

In the present study, it was observed that each nerve contained a different number of fascicles and these fascicles were different both in size and in the number of axons they contained. All these variables could be the reason why the desired outcomes cannot always be achieved in nerve reconstruction.

## Highlights

• The amount of interfascicular connective tissue in sensory and motor nerves.
• Which nerve graft seems more logical for facial nerve anastomosis.
• Axons number of great auricular nerve.

## Introduction

The history of nerve repair, which aims to restore the functions of severed or damaged nerves, dates back to the late 19th century. Hulter identified the epineural suture technique in 1873, and Nicholson and Seddon reported the first functional successful median nerve reconstruction using a similar technique in 1957.^1^ In 1960, Millesi used surgical microscope in nerve reconstruction, and Smith reported the successful use of microscope in 1964, paving the way for microsurgery in this area.^2^

The main purpose of nerve repair is to ensure anatomical continuity of as many axons as possible. Epineural suturing is a classical surgical reconstruction technique but may not be able to provide interfascicular connection. Therefore, the interfascicular suturing technique has been refined to ensure interfascicular connection with the continuity of as many axons as possible. This technique also allows the application of monofascicular and polyfascicular nerve grafts.^3^ However, the question of whether each fascicle in a nerve contains the same number of axons has needs to be answered first. Therefore, it is important to know the number of axons as well as the number of fascicles in the nerves to be used for grafting purposes.

The extratemporal part of the facial nerve can be damaged in facial injuries and during parotid tumor surgeries.^4^, ^5^, ^6^ Most surgeons mention that primary nerve reconstruction is the best option for axonal regeneration in repairs after nerve damage.^7^, ^8^ However, primary nerve reconstruction is rarely possible. An autologous neural graft, which acts as a bridge, is required to ensure nerve continuity.^9^ The most commonly used nerves for this purpose are hypoglossal nerve, great auricular nerve (GAN) and the sural nerve.^10^

The aim of the present study was to examine three nerves most commonly used for grafting purposes after facial nerve damage, i.e., hypoglossal nerve, GAN and sural nerve, and to determine their thickness, fascicle area, and fascicle and axon numbers. Thus, the nerves which are more suitable for grafting purposes were investigated.

## Methods

The study was carried out in Ear-Nose-Throat and Forensic and Histology clinics of a tertiary health-care facility. The local ethic committee approved the study (Approval nº 18-KAEK-077). Autopsies involving situations such as crushing and severing that would impair nerve integrity in the face, neck, and leg area from which the samples would be taken were excluded. Autopsies involving peripheral neurologic and/or neuropathic disease were also excluded. The relatives of the autopsied individuals from which the nerve samples would be taken were informed about the procedures to be carried out and their consent was obtained. The procedures were requested to be completed within a maximum of two hours by the relatives of the autopsies due to traditional and religious reasons to ensure that burials were not disrupted, and by the officers on duty to ensure that their work was not delayed. All autopsy dissections were performed within the first 24 h after death.

### Dissecting nerves

To dissect the facial nerve, hypoglossal nerve and GAN during autopsy, the incision from the ear lobe to the sternal notch, one of the routine autopsy incisions, was used for each side of the neck. For the aim of a more comfortable access to the facial nerve, the incision in the ear lobe was extended about 1 cm upwards towards the tragal cartilage when necessary. Skin, subcutaneous tissues and platysma muscle were divided on both sides of the neck. The exposed GAN sample was taken from the 1/3 middle course after emerging from posterior border of sternocleidomastoid muscle (SCM) and before anterior and posterior branching.^10^ During the search for the facial nerve, tragal cartilage was first dissected and the tragal pointer was found. Then the posterior belly of the digastric muscle was found. The dissection was continued from the mastoid process medially and anterior. The main segment of the extracranial facial nerve was found approximately 1–1.5 cm anteroinferomedial to the tragal pointer.^11^ Dissection was continued and the truncus temporofacialis and truncus cervicofacialis were found, followed by the rami.^11^ Nerve samples were taken after all the facial nerves and branches were exposed. To locate the hypoglossal nerve, the internal jugular vein was found in the medial part of the SCM muscle and the main carotid artery was found. Above the carotid triangle, the submandibular gland was dissected upward. In the superior of the hyoid bone, the hypoglossal nerve was found to cross these two arteries during dissection of the internal and external carotid arteries.^12^ Sampling was made from the portion of the hypoglossal nerve before branching.^12^ In order to dissect a sural nerve sample, a 3 cm-vertical incision was made on about 1.5–2 cm posterior of lateral malleolus. After the skin and subcutaneous tissues were crossed, sural nerve and vena saphena parva translucence were observed deep in the fascia plane.^13^ Dissection was continued, and sural nerve was reached. A sural nerve sample was taken in the section adjacent to *v. saphena parva* between Achilles’ tendon and lateral malleolus.^13^

### Histological evaluation

**Figure 1:**
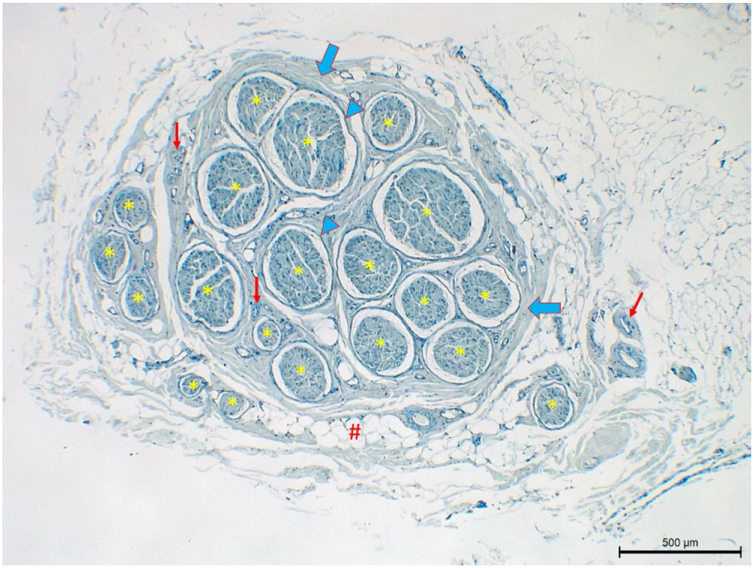
Left nervus auricularis magnus with 20 fascicles (*: fascicule, thick blue arrow: epineurium, thick blue arrowhead: perineurium, thin red arrow: vessel; #: adipose tissue).

**Figure 2:**
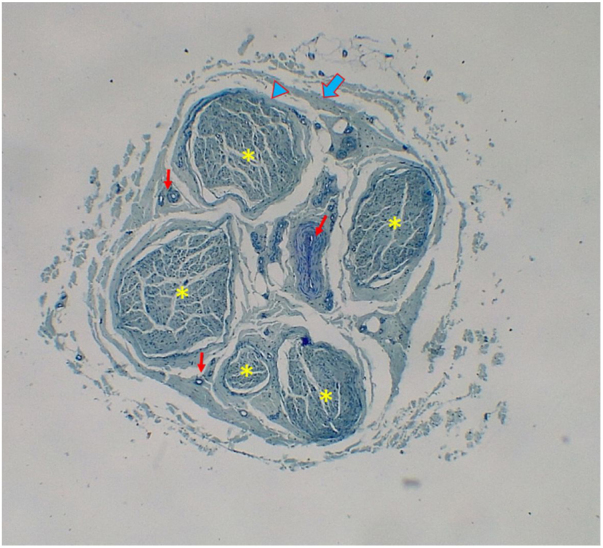
Right nervus suralis with 5 fascicles (*: fascicule, thick blue arrow: epineurium, thick blue arrow head: perineurium, thin red arrow: vessel).

**Figure 3:**
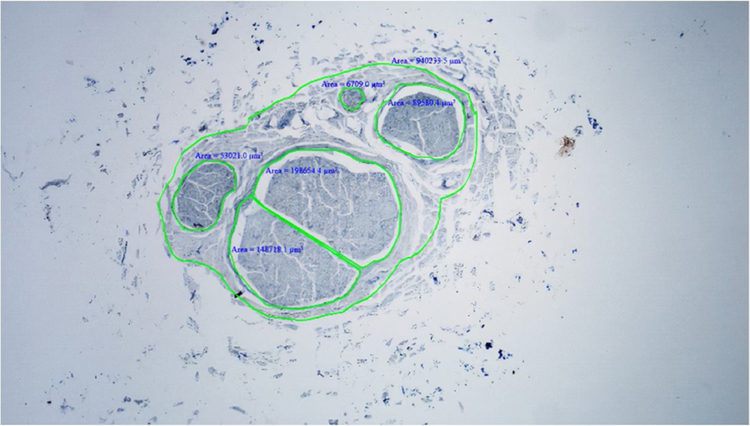
Calculation of nerve and fascicle areas.

**Figure 4:**
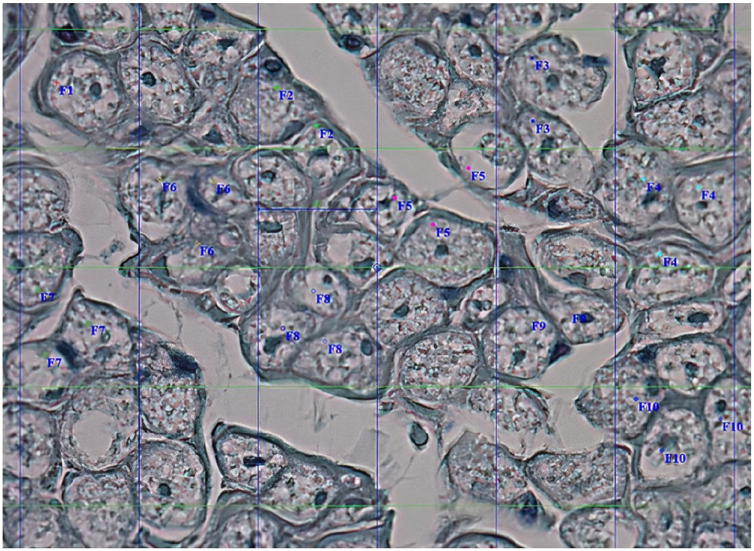
Calculation of the number of axons.

Neural fiber specimens of each individual were observed micromorphometrically under the light microscope (Nikon Eclipse 200; Nikon Corp., Tokyo, 108–6290, Japan) in the cross-sectional preparations using NIS-Element software (Hasp ID: 6648AA61; Nikon Corp, Tokyo, 108–6290, Japan) (Figure 1, Figure 2). The outmost boundaries of the epineuriums were outlined in a line with the help of the system and nerve fiber diameters were calculated micrometrically in quadruple lens enlargement. In addition, the perineurium connective tissue covers around each fascicle were surrounded by a line using the software, and the diameter of each fascicle was calculated separately (Fig. 3). The number of axons was calculated by counting axons in the 100× lens images of each fascicle transferred to the monitor via the camera. For this purpose, a counting frame of 20 × 20 μm = 400 μm^2^ was placed randomly on these images. With this counting frame, the axons in 10 different counting frame areas, placed systematically and randomly, were marked, and counted by the system (Fig. 4).^14^ The total number of axons obtained was divided by 10 and the average number of axons per unit area was found.

### Statistical analyses

Statistical analyses of the data were conducted using SPSS software (ver. 20.0, IBM Inc., NY 10504–1722, USA). Independent sample *t*-test was used when the data fulfilled the parametric assumptions. When the data did not fulfill the parametric assumptions, Mann–Whitney *U* and Kruskal–Wallis Test were used; p-values smaller than 0.05 were considered statistically significant.

## Results

Consent was obtained from the family members of 12 out of the 22 cadavers suitable for taking nerve samples. All individuals from whom the neural samples were taken were male. The mean age was 45.8 ± 12.6 years (range: 28–64).

For facial nerve extracranial segment, six samples were dissected from the right side and eight from the left. The number of samples taken from other facial nerve trunk and branches are given in Table 1. For the sural nerve, 10 samples were taken from the right side and 8 from the left. For GAN, 11 samples were taken from the right side and 12 from the left. Finally, four samples of hypoglossal nerve were taken from both left and right sides.

**Table 1:** Number of nerves taken from autopsy samples and number of fascicles in each nerve.

Nerve number taken		1	2	3	4	5	6	7	8	9	10	11	12
Facial nerve extracranial main segment	Right	6	3	7	8	6	19						
	Left	5	3	7	9	5	19	11	4				
Truncus temporofacialis	Right	10	13	7	5	9	7						
	Left	11	17	8	5	9	7	4	15				
Truncus cervicofacialis	Right	2	5	15	10	4	2						
	Left	5	5	13	8	7							
Ramus temporalis	Right	2	6	6	1	3	2						
	Left	2	4	7	1	3	4	8					
Ramus zygomaticus	Right	5	4										
	Left	3	3										
Ramus buccalis	Right	2	11	6	4	4	6						
	Left	3	10	6	7	5	9						
Ramus marginalis mandibularis	Right	4	11	2	2	7	5						
	Left	6	8	2	2	6	6						
Ramus cervicalis (left)		3	3	8									
Nervus suralis	Right	6	14	7	9	17	11	10	10	5	3		
	Left	6	14	8	13	18	14	10	15				
Nervus auricularis magnus	Right	18	3	18	20	4	15	17	6	6	16	7	
	Left	21	4	24	20	8	11	14	5	4	9	9	3
Nervus hypoglossus	Right	7	4	5	6								
	Left	4	4	3	6								

Since two nerves can be taken on each side of the ramus zygomaticus and only three samplings could be made on the left side in the ramus cervicalis, no comparisons could be made between the right and left sides of these two nerves. There was no significant difference between the right and left sides of other nerves in terms of nerve area, fascicle area, number of fascicles and average number of axons (*p* > 0.05) (Table 2, Table 3).

**Table 2:** Nerve and fascicle areas, and fascicle and axon numbers in left and right sides of facial nerve and its branches.

Name of the nerve		Average nerve area (µm^2^)	Average fascicle area (µm^2^)	Average fascicle number	Average axon number
Facial nerve extracranial main segment	Right (n = 6)	2,463,980 ± 1,330,110	1,195,581 ± 214,054	8.2 ± 5.6	12,370 ± 2315
	Left (n = 8)	2,474,122 ± 1,637,003	1,253,126 ± 697,568	7.9 ± 5.2	13,601 ± 4242
p		0.930	0.831	0.923	0.502
Truncus temporofacialis	Right (n = 6)	1,905,340 ± 525,875	765,370 ± 226,236	8.5 ± 2.8	6947 ± 1789
	Left (n = 8)	2,106,935 ± 957,511	653,102 ± 232,884	9.5 ± 4.6	5811 ± 1874
p		0.652	0.384	0.625	0.274
Truncus cervicofacialis	Right (n = 6)	960,499 ± 422,809	330,071 ± 187,556	6.3 ± 5.2	2908 ± 1403
	Left (n = 5)	1,103,741 ± 332,496	407,110 ± 162,311	7.6 ± 3.3	3718 ± 1340
p		0.554	0.490	0.635	0.356
Ramus temporalis	Right (n = 6)	672,434 ± 393,258	282,447 ± 144,644	3.3 ± 2.2	2532 ± 1093
	Left (n = 7)	970,530 ± 59,9256	358,244 ± 185586	4.1 ± 2.5	3340 ± 1779
p		0.321	0.435	0.548	0.340
Ramus zygomaticus	Right (n = 2)	980,705 ± 571,426	425,107 ± 248,495	4.5 ± 0.7	3188 ± 1864
	Left (n = 2)	926,414 ± 165,131	388,292 ± 110,750	3.0 ± 0.0	3338 ± 228
Ramus buccalis	Right (n = 6)	918,706 ± 793,607	251,540 ± 151,999	5.5 ± 3.1	2386 ± 1368
	Left (n = 6)	740,200 ± 194,349	220,009 ± 92,788	6.7 ± 2.6	2344 ± 1115
p		0.604	0.676	0.494	0.955
Ramus marginalis mandibularis	Right (n = 6)	823,153 ± 502,648	221,844 ± 146,033	5.2 ± 3.4	2523 ± 2030
	Left (n = 6)	912,099 ± 545,909	255,737 ± 136,389	5.0 ± 2.4	2757 ± 1705
p		0.528	0.687	0.925	0.834
Ramus cervicalis left (n = 3)		804,183 ± 80,820	199,291 ± 278,165	4.7 ± 2.9	1619 ± 1243

**Table 3:** Nerve and fascicle areas, and fascicle and axon numbers in left and right sides of nervus hypoglossus, nervus suralis and nervus auricularis magnus.

Name of the nerve		Average nerve area (µm^2^)	Average fascicle area (µm^2^)	Average fascicle number	Average axon number
Nervus auricularis magnus	Right (n = 11)	1,906,016 ± 719,312	489,255 ± 298,183	11.8 ± 6.5	4394 ± 1731
	Left (n = 12)	1,926,849 ± 853,239	538,279 ± 199,883	11.0 ± 7.2	4492 ± 1677
p		0.950	0.652	0.778	0.891
Nervus suralis	Right (n = 10)	2,160,042 ± 1,265,801	599,636 ± 257,929	9.2 ± 4.2	5193 ± 2453
	Left (n = 8)	2,222,540 ± 881,637	606,853 ± 139,400	12.3 ± 4.0	5137 ± 1311
*p*		0.904	0.941	0.134	0.951
Nervus hypoglossus	Right (n = 4)	3,160,921 ± 1,066,926	1,621,197 ± 501,321	5.5 ± 1.3	14,921 ± 5338
	Left (n = 4)	3,204,655 ± 707,561	1,525,164 ± 480,156	4.3 ± 1.3	14,623 ± 4081
*p*		0.948	0.791	0.215	0.932

The lowest mean number of fascicles was found in the hypoglossal nerve (4.9 ± 1.4), while the highest was in GAN (11.4 ± 6.8) (Table 4). However, it was found that the number of fascicles in the nerves was quite variable. It was observed that the number of fascicles of the same nerve can be quite variable among individuals. For example, three fascicles were detected in one facial nerve sample while another had 19 fascicles. Similarly, 24 fascicles were observed in a GAN sample while another had only three (Table 1). However, the variation for the total number of axons in one nerve among different individuals was less than the variation for the number of fascicles (Table 2, Table 3, Table 4).

**Table 4:** Nerve and fascicle areas, and fascicle and axon numbers of the nerves.

Name of the nerve	Average nerve area (µm^2^)	Average fascicle area (µm^2^)	Average fascicle number	Average axon number
Facial nerve extracranial main segment (n = 14)	2,469,776 ± 1,457,204^a^	1,228,464 ± 529,635^c^	8.0 ± 5.2^g^	13,074 ± 3485^1,2^
Truncus temporofacialis (n = 14)	2,020,538 ± 781,510^a,b^	701,217 ± 228,502^c,e^	9.1 ± 3.8^f,g^	6298 ± 1861^1,3,4^
Truncus cervicofacialis (n = 11)	1,025,609 ± 373,097^a^	365,089 ± 172,468^c^	6.9 ± 4.3^g^	3276 ± 1371^1,5,6^
Nervus hypoglossus (n = 8)	3,182,788 ± 838,430 ^a^	1,573,181 ± 457331^c^	4.9 ± 1.4^f,g^	14,772 ± 4402^2,3,5,7^
Nervus auricularis magnus (n = 23)	1,916,885 ± 774,150^a,b^	514,833 ± 47,019^c,δ^	11.4 ± 6.8^g^	4445 ± 1665^1,6,7,8^
Nervus suralis (n = 18)	2,187,819 ± 1,081,356^a,b^	602,844 ± 207,931^c,d,e^	10.6 ± 4.3^g^	5168 ± 1973^1,4,7,8^
*p*	0.000	0.000	0.014	0.000

The difference among the groups for average nerve area is significant = ^a^ (*p* < 0.05); not significant = ^b^ (*p* > 0.05).

The difference among the groups for average fascicle area is significant = ^c^ (*p* < 0.05); not significant = ^d,e^ (*p* > 0.05).

The difference among the groups for average fascicle number is significant = ^f^(*p* < 0.05); not significant = ^g^ (*p* > 0.05).

The difference among the groups for average axon number is significant = 1, 3, 5, 7 (*p* < 0.05); not significant = 2, 4, 6, 8 (*p* > 0.05).

Rami of the facial nerve were not included in the comparisons among the nerves because they had lower thickness, fascicle area and number of axons compared to other nerve samples used for grafting purposes. There was no significant difference among truncus temporofacialis, GAN and nervus suralis for the nerve area (p > 0.05). The thickness of truncus temporofacialis was almost twice the thickness of truncus cervicofacialis.

The thickness of the extracranial main segment of facial nerve was slightly higher than that of GAN and sural nerve (*p* < 0.05), and the difference was much more prominent for fascicle area and number of axons (*p* < 0.05). Although the fascicle area of the extracranial main segment of fascial nerve was slightly less than that of the hypoglossal nerve, there was no significant difference between the two nerves for axon numbers (*p* > 0.05). Hypoglossal nerve was thickest of all nerves (3,182,788 ± 838,430 μm^2^) and had the highest fascicle area (1,573,181 ± 457,331 μm^2^) and number of axons (14,772 ± 4402) (*p* < 0.05) (Table 4). The number of axons per unit nerve area (axon count/nerve area) was significantly higher in motor nerves (facial nerve 5293 mm^2^, truncus temporofacialis 3116 mm^2^, truncus servicofacialis 3194 mm^2^ and hypoglossal nerve 4641 mm^2^) compared to sensory nerves (sural nerve 2362 mm^2^ and GAN 2318 mm^2^) (p < 0.05). The number of axons per unit fascicle area (number of axons/fascicle area) was also higher in motor nerves than in sensory nerves. This difference was significant in the main segment of the facial nerve (10,642 mm^2^) and hypoglossal nerve (9389 mm^2^) compared to sensory nerves of sural nerve (8572 mm^2^) and GAN (8633 mm^2^) (*p* < 0.05). In addition, the number of axons in the facial nerve and hypoglossal nerve, which have motor fibers, were significantly higher than in GAN and sural nerve, which have sensory fibers (*p* < 0.05) (Table 4).

## Discussion

The key to a successful nerve reconstruction is a thorough understanding of the microanatomy of the nerves to be repaired or used as grafts. Thus, it is important to know the microanatomy of the facial nerve, which has an important place in Ear–Nose–Throat practice, and of the nerves used for grafting purposes in its repair.

In their study on 25 cadavers, Guerrissi and Miranda^3^ found that thickness of the extratemporal main trunk of the facial nerve varied between 1000 and 1400 μm (range: 800–1600 μm) in the majority of cases and that the number of fascicles was two or three in most cases (range: 2–10). The thickness of superior and inferior branches, on the other hand, varied between 800 and 1200 μm (range: 600–1500 μm) in most cases, and fascicle number varied between two and seven (range: 1–13) in the majority of cases. In the present study, the measurement was performed not with calipers, but by field measurement under the microscope. Therefore, the comparison was difficult. However, considering the nerve as round and taking an average facial nerve diameter (2 r) of 1200 μm, the nerve area could be calculated as 1,130,400 μm^2^.^3^ This figure was about half of the nerve area measured in the present study. The number of fascicles was determined to be 8.0 ± 5.2 in the main trunk, 9.1 ± 3.8 in temporofacial trunk and 6.9 ± 4.3 in the cervicofacial trunk in the present study, which were somewhat higher than the values reported by Guerrissi and Miranda.^3^ Captier et al.^4^ examined 10 cadavers ranging in age from 42 to 85 measuring the fascicle size rather than histologically measuring the nerve area, and found that the total fascicle size of the facial nerve was 690 μm in the extratemporal segment, 560 μm in the temporofacial branch and 480 μm in the cervicofacial branch. They also observed that the number of fascicles in the main trunk of the facial nerve could be quite variable (between 2 and 15) and that the number of fascicles increased at the point of separation into branches. The average number of fascicles was 13 (range: 2–29) in the temporofacial branch and 16 (range: 2–23) in the cervicofacial branch. The average number of axons was 6490 ± 1092 in the main trunk of the facial nerve, 4264 ± 1905 in temporofacial branch and 1774 ± 1017 in the cervicofacial branch. The number of axons in the temporofacial branch was notably higher than that in the cervicofacial branch.^4^ Guerresi and Miranda^3^ did not determine the nerve size, fascicle area and the number of axons. Therefore, it may not be correct to compare the fascicle size measurement made by Captier et al.^4^ with the fascicle area measurement in the present study because nerves have round-like shapes while the shape of the fascicles can be extremely variable ranging from round to extremely oval or ellipse-shaped. The number of axons determined in the present study was almost twice of what was reported by Captier et al.^4^

Guerresi and Miranda^3^ reported that in the main trunk of the facial nerve, most of the axons are located in several large fascicles, and these areas may be more sensitive to trauma. In these parts, there is even less amount of perifascicular tissue that could protect the nerve from crush-type trauma. Since there are several large fascicles in these parts, it seems appropriate to perform microsurgical repair with epineural suturing to ensure a proper continuity of most axons.^3^ Because of the polyfascicular distribution in the truncus temporofacialis and truncus cervicofacialis, repair with interfascicular suture appears to be the ideal reconstruction technique, and a polyfascicular nerve graft can be used for this aim. Since the connective tissue is abundant in these areas, excessive connective tissue should be excised before suturing the fascicles during the repair. If not excised, this tissue can multiply during healing and invade the surrounding area.^3^ Similarly, in the present study, it was observed that the fascicle area of the main trunk of the facial nerve is about half of the nerve area, while in truncus temporofacialis and truncus cervicofacialis fascicles occupy almost a third of the nerve area. In addition, fascicular area is about half of the nerve area in the facial nerve and hypoglossal nerve, while in the GAN and sural nerve fascicles constitute about one-third of the nerve area. Consequently, it can be stated that the amount of interfascicular connective tissue is higher where nerve fiber progresses and splits into branches, and that sensory nerves contain more connective tissue than motor nerves.

Research on the microanatomy of hypoglossal nerve is limited.^15^, ^16^, ^17^, ^18^ Asaoka et al.^15^ evaluated both facial and hypoglossal nerves on 12 sides of 7 adult cadavers. They also took samples after vestibular schwannoma surgery from three patients who had facial paralysis and who underwent hypoglossal-facial side-to-end anastomosis.^15^ The hypoglossal nerve was detected as monofascicular at atlas level, and its thickness in this region was 1.541 ± 0.332 mm^2^ (range: 1.012–2.34).^15^ They found the number of axons in the hypoglossal nerve as 9778 ± 1516 (range: 7654–12,458). Unlike the present study, facial nerve sampling was made only from the tympanic segment in that study. It was revealed that the facial nerve was monofascicular until around the chorda tympani branch, after which several small fascicles began to form.^15^ It was revealed that in the tympanic segment, the thickness of the facial nerve was 61.5% and number of axons was 73.2% compared to the hypoglossal nerve. Another noteworthy finding in that study was that damaged facial nerves were more atrophic and thinner than the normal nerves.

Samii and Matthies^19^ emphasized that compatibility of the nerve fields between donor and receiver nerves is technically important in successful nerve repair. Asaoka et al.^15^ reported that since the facial nerve area is smaller than the hypoglossal nerve area, hypoglossal nerve can be split into two vertically, and one part can be used for facial nerve repair while the other is preserved as a continuation of the function of the tongue muscles. Mackinnon and Dellon^16^ dissected the submandibular triangle in 10 cadavers and named the hypoglossal nerve “proximal” in the immediate distal of ansa hyoglossi, “middle” in the section under the posterior digastric muscle, and “distal” in the section just before it enters into the mylohyoid muscle. Monofascicular structure was observed in almost the entire hypoglossal nerve until the distal part. The number of fascicles was found to be 1.1 ± 0.35 in the proximal, 1.1 ± 0.38 in the middle and 5.0 ± 4.5 in the distal sections. The number of fascicles in the distal section was statistically higher than in other segments (*p* < 0.01). The average number of axons in the hypoglossal nerve was 9202 ± 2182,^16^ and the extracranial part of the hypoglossal nerve was observed to switch from the monofascicular to polyfascicular pattern as it moves towards the distal in the submandibular region.^16^ Hence, interfascicular anastomosis does not seem possible in the proximal part of the hypoglossal nerve. In this part, it was thought that splitting the nerve longitudinally could cause axon damage. The area sampled in the present study corresponded to a place between the middle and distal sections defined by Mackinnon and Dellon.^16^ We took the nerve sample here in the section before the nerve produces a branch leading to the inferior. Maximum care was observed to take samples in the same place since the neural parameters could vary in different regions. The average number of fascicles in the present study was 4.9 ± 1.4, which was similar to what was reported previously.^16^ The number of axons, on the other hand, was 14,772 ± 4402 and this number was slightly higher than that reported by Mackinnon and Dellon.^16^ As in other studies^15^, ^16^, ^20^, ^21^ both size and number of axons in the hypoglossal nerve were greater than the extemporal main segment of the facial nerve in the present study.

Since the number of axons per unit nerve area and unit fascicle area is similar in motor nerves, the use of hypoglossal nerves in facial nerve anastomosis seems more logical. However, it is important to consider the motor deficit risks that may occur in patient and share these risks with the patient. Conley^17^, ^18^ found that 74% of patients who underwent hyoglossi-facial anastomosis had functional problems, and 21% had severe swallowing difficulties. Conley split the hypoglossal nerve vertically to overcome these previously known problems,^17^ and in another attempt used the inferior half of the hypoglossal nerve but was not successful.^18^ May et al.,^22^ on the other hand, used an interposition graft between the hypoglossal nerve and the distal part of the facial nerve through opening a window in the hypoglossal nerve, claiming minimal tongue morbidity in only 4% of patients. The presence of a topographical localization in the hypoglossal nerve related to innervation of the tongue muscles is another consideration that needs to be addressed. It was found that in the 1/3 distal side of the nerve, posterior fascicles innerve the posterior muscles of the tongue, which is more related to swallowing and aspiration.^16^ Thus, it seems beneficial to evaluate the hypoglossal nerve intraoperatively and to use the anterior and cephalic parts in nerve anastomosis, preserving posterior tongue functions, as indicated by Mackinnon and Dellon.^16^

There are some studies in the literature examining the number of fascicles in *n. suralis*^23^, ^24^, ^25^ but no study has investigated the number of axons so far. The nerve thickness varied from about 2–4 mm while the number of fascicles ranged between 2 and 14.^23^, ^24^, ^25^ In another study, fascicle area was measured as 0.43 mm^2^ in the middle calf area and 0.55 mm^2^ in the lower calf area.^23^ All of the sural nerve samples in the present study were taken from about 1–1.5 cm posterior of lateral malleol. It is recommended that the nerves to be used for grafting purposes should be taken from this location in that no variations are observed, and complication risk is low.^26^ In the present study, nerve thickness was 2.2 ± 1.1 mm^2^ and fascicle area was 0.6 ± 0.2 mm^2^. The number of fascicles was 10.6 ± 4.3. Area and number of fascicles determined in the present study were higher than what was found by Park et al.^23^ Nerve thickness in the present study was similar to other studies.^24^, ^25^ We observed that the sural nerve size was slightly smaller than the main trunk of the facial nerve and slightly larger than truncus temporofacialis. The number of axons, on the other hand, was considerably less than that of facial nerves, and slightly less than that of truncus temporofacialis. In terms of number of fascicles, it was revealed that all three nerves were multifascicular. Therefore, it could be stated that the sural nerve could be used for grafting purposes in acute damage to truncus temporofacialis. It is known that the nerve thickness and number of axons decrease in the long-term damage to the facial nerve.^15^ Therefore, it could be more appropriate to use the sural nerve for grafting purposes in chronic, rather than acute, damage to the facial nerve.

While there are some studies in the literature dealing with the nerve thickness, fascicle area and number of fascicles of GAN,^10^, ^27^, ^28^, ^29^, ^30^ the number of axons has not been studied so far. Altafulla et al.^10^ examined 11 sides in 6 cadavers and found that the average GAN thickness was 1.51 ± 0.23 mm (range: 1.11–1.91) in distal, 1.38 ± 0.34 mm (range: 0.66–1.88) in middle and 1.58 ± 0.26 mm (range: 1.01–1.97) in proximal sections. Similar to our findings, Altafulla et al.^10^ found no difference between the sides in terms of size and thickness (*p* > 0.05). Yang et al.^28^ examined GAN in 25 sides of 14 cadavers with an average age of 62.5 years. They cross-sectioned and histologically examined the GAN proximally just after SCM and distally just before branching. They found the average number of fascicles to be 2.5 (range: 1–4) in the proximal and 5 (range: 2–8) in the distal. They reported the total fascicle area as 1.42 mm^2^ (range: 0.84–1.75) in the proximal and 0.6 mm^2^ (range: 0.54–0.64) in the distal. While the number of fascicles in GAN increased from proximal to distal, a decrease was observed in fascicle area.^28^ On the other hand, we made the nerve sampling right in the midpoint of GAN after it emerged from posterior of SCM but before branching, i.e., in the middle section. In order to compare with the findings of Altafulla et al.,^10^ nerve area in their study was calculated as 1.50 mm^2^ using the nerve diameter of roughly 2 r: 1.38 mm in the middle area. On the other hand, we found this area as 1.92 mm^2^, i.e., somewhat larger. The fascicle area found by Yang et al.^28^ in the distal was similar to the area in the present study, but what they found in the proximal was larger than our values. The number of fascicles in the present study, on the other hand, was higher than what Yang and et al.^28^ found both in the proximal and distal. The reason why the fascicle area in the present study was lower than what Yang et al.^28^ reported was the higher number of fascicles in the present study and consequent increase in the interfascicular connective tissue, which could reduce the fascicle area.

In the present study, we aimed to eliminate possible differences in nerve area, fascicle area, number of fascicles and number of axons by taking each nerve sample from the same localization because peripheral nerve structure can vary throughout the trace. As the nerve progresses, the number of fascicles can increase or they can merge, thereby increasing the size of the fascicles. It can be divided into side branches or combined with the collaterals.^3^ Another important consideration in the present study was that all nerve samples were taken from all individuals. Thus, we aimed to reduce the variability that may have occurred in the facial nerves and grafts of different individuals. However, we experienced technical difficulties and time constraints due to the reasons such as the difficulty with turning the head and feet of the cadaver as a result of rigor mortis, rushing by the family members of the cadaver for burials, and unwillingness of the individuals officially involved in the autopsy process to extend their working hours. Accordingly, there were cases where we were not able to make all the samplings from a cadaver. We also could not find proper female cadavers. Some cadaver’s relatives did not give permission for dissection, thus we cannot give any results of female cadaver’s nerve information. In optimum conditions, we had the objectives of performing all samplings from a cadaver and even sampling from different localizations of hypoglossal nerve, sural nerve, and GAN for both genders.

## Conclusion

It was revealed in the present study that each nerve contains a different number of fascicles, and these fascicles vary both in size and in the number of axons they contain. In addition, it was observed that the number of axons per unit nerve area and per unit fascicular area could be different. We hypothesize that all these variabilities are among the reasons for the failure to uniformly get satisfactory results in nerve reconstruction.

## Funding

No funding was received for this work.

## Conflicts of interest

The authors declare no conflicts of interest.
